# Cortisol, cardiovascular risk, and anxiety in full-time workers in Cartagena, Colombia, 2023

**DOI:** 10.3389/fpsyt.2025.1491987

**Published:** 2025-04-10

**Authors:** Alicia Norma Alayón, Nohora Ochoa Arizal, Manuel Noreña Correa, Jose López Toro, Francisco Hernández Rojas

**Affiliations:** ^1^ Biomedical Research Group, Faculty of Health Sciences, Bacteriology Program, Universidad de San Buenaventura, Seccional Cartagena, Cartagena, Colombia; ^2^ Psychology Research Group, Faculty of Human and Social Sciences, Psychology Program, Universidad de San Buenaventura, Seccional Cartagena, Cartagena, Colombia; ^3^ Planning Department, Universidad de San Buenaventura, Seccional Cartagena, Cartagena, Colombia

**Keywords:** anxiety state, anxiety trait, cardiovascular risk, cortisol, circadian rhythm

## Abstract

**Background:**

Considering the general trend toward an increased occurrence of cardiovascular and mental health diseases, we studied the relationship between the presence of trait and state anxiety and the alteration of serum cortisol, lipid, and glycemia levels.

**Material and methods:**

The study assessed 90 full-time workers waist circumferences, as well as their cortisol levels at 8 AM and 4 PM, and their fasting serum glucose and lipid profiles. The construct of trait and state anxiety was assessed by means of the Inventory of Trait-State Anxiety (IDARE Spanish version).

**Results:**

The state anxiety scale showed high reliability (ω = 0.94, α = 0.939). Moderate to high state anxiety was observed in 61.1% of participants, while 71.1% had moderate to high trait anxiety. Most participants (94.4%) had AM and PM cortisol levels within normal ranges. PM cortisol levels were lower than AM cortisol levels in 95.6% of participants (86/90). Dyslipidemia was present in 60.4% of participants. No significant sex differences were found in AM and PM cortisol or anxiety levels, except for triglycerides, which were higher in men (p = 0.013). State anxiety was positively correlated with PM cortisol levels (r = 0.232, p = 0.028), no significant associations were found with AM cortisol or age. A significant association was observed between waist circumference and fasting glycemia, with 68.9% of participants exceeding the recommended waist circumference threshold. Obesity was significantly associated with hyperglycemia (p = 0.010). An ANOVA revealed a significant effect of state anxiety on evening cortisol levels (F(2, 87) = 7.336, p = 0.001), with the high state anxiety group exhibiting the highest PM cortisol levels. Additionally, a t-test found a significant difference in triglyceride levels between the presence and absence of state anxiety t (87.999) = -2.244, p = 0.027.

**Conclusions:**

The presence of state anxiety proved to be the type of anxiety most associated with increased evening cortisol levels and triglyceride levels. Understanding the relationships between mental states and biochemical physical conditions will be essential in the future for maximizing the benefits of technological developments applied to the diagnosis, prognosis, and monitoring of patients’ overall health.

## 1 Introduction

Mental health is a significant component of the well-being of human beings. The World Health Organization describes it as a state of well-being in which individuals exercise their abilities, cope with the stresses of life, work productively and fruitfully, and give something to their community (1). Mental disorders cause alterations in individuals’ thoughts, emotions, and behaviors, which interfere with their quality of life and functionality (2). Additionally, mental disorders are associated with different medical conditions. They may affect their etiology or be correlated or coexist with physical diseases, for example, cardiovascular conditions, diabetes, infectious diseases, cancer (3), and chronic pain (4).

Anxiety disorders are the most prevalent mental disorders in the world; in 2019, they affected 301 million people (5). In Colombia, the 2015 National Survey of Mental Health conducted in adults reported a prevalence of 1.6% for major depression, 0.3% for generalized anxiety, and 2.1% for any anxiety disorder (6). It can be seen that anxiety, in particular, is one of the most frequent health concerns in the world and Colombia, so actions for its prevention and effective treatment are needed.

Clark and Beck (7) define anxiety as “a behavioral, physiological, affective, and cognitive response to events that are perceived as threats to vital interests and as uncontrollable or unpredictable.” These authors differentiate anxiety from fear by considering the latter as a primitive automatic neurophysiological response to perceived imminent dangers to physical or psychological safety.

Some common physical symptoms of anxiety are tremors in the body or hands, hot flashes, chills, palpitations, dry mouth, sweating, shortness of breath, chest pain or pressure, and muscle tension (8). Moreover, the neuroendocrine or metabolic basis of anxiety initially has an adaptive purpose, as it physically prepares the organism to face a possible threat. This is evident in some hyperactivation signs triggered by anxiety, such as peripheral blood vessel constriction, increased skeletal muscle strength, increased heart rate and lung contraction and expansion capacity to promote oxygenation, dilation of the pupils to improve vision, reduced digestive activity, increased basal metabolism, and increased epinephrine and norepinephrine release from the adrenal medulla (7).

Faculty members are not exempt from anxiety-related phenomena and, in fact, may be exposed to particularly anxiety-inducing situations. Research has shown that academic environments present unique stressors that contribute to faculty distress, including workload, performance pressures, and student interactions. A study conducted at West Visayas State University identified paperwork and administrative responsibilities as primary sources of stress among faculty, with high blood pressure and anxiety reported as common physiological and emotional consequences (9). Similarly, Filaire et al. (10) found that lecturing to a large audience led to significant increases in salivary cortisol and alpha-amylase, indicating heightened activation of the hypothalamic–pituitary–adrenal (HPA) axis and sympathetic-adrenal-medullary (SAM) system. These physiological changes were accompanied by elevations in pro-inflammatory cytokines, suggesting an immune response to acute psychological stress. Additionally, Sabagh et al. (11) highlighted that faculty burnout is strongly associated with excessive job demands, value conflict, and lack of institutional support, leading to adverse psychological and physical health effects, such as depression and reduced job performance. These findings collectively underscore the impact of chronic and acute stress in academia, reinforcing the need for institutional strategies to mitigate anxiety and cortisol dysregulation among faculty members.

More generally, given that anxiety is the most prevalent mental disorder, it correlates with a wide range of physical diseases; for example, anxiety correlates with endocrine disorders, including diabetes mellitus, thyroid disease, and catecholamine-producing neuroendocrine tumors like pheochromocytomas (12). It is also associated with gastrointestinal disorders such as peptic ulcers, celiac disease, and irritable bowel syndrome. In addition, it is associated with musculoskeletal disorders such as fibromyalgia/chronic fatigue syndrome and arthritis, as well as neurological disorders such as migraines, epilepsy, and neurodegenerative diseases. Cardiorespiratory diseases such as asthma, angina pectoris, chronic obstructive pulmonary disease, mitral valve prolapse, cystic fibrosis, and obesity also show a relationship with anxiety. Furthermore, chronic pain, including burns and cancer, as well as infectious diseases such as human immunodeficiency virus and tuberculosis, may be exacerbated by anxiety (3). The relationship between mood and metabolic dysfunctions may perpetuate the cycle (13).

In this study, we use the concepts of trait anxiety and state anxiety put forward by Spielberger et al. (14). On the one hand, “trait anxiety” refers to constant anxiety experienced by individuals and is identified as a personality trait, so it is more stable and permanent over time. On the other hand, “state anxiety” is considered a transitory emotional state occurring in response to a particular stressful situation. These concepts are crucial to understanding how external and internal (e.g., cognitive) factors can trigger anxiety (8).

One of the metabolic responses related to coping with a stressful situation is the secretion of cortisol, a cholesterol-derived hormone and the main glucocorticoid produced in the cortex of the adrenal glands, located in the upper part of both kidneys. Its main functions include lipid reorganization and metabolism, as well as insulin secretion, blood pressure increase, and suppression of the immune system as an anti-inflammatory mechanism (15).

When released, it can inhibit the hypothalamic release of corticotropin-releasing hormone and pituitary release of adrenocorticotropic hormone, known as negative feedback. Under persistent stress conditions, this negative feedback loses effectiveness, and high cortisol levels may remain for a long time, so it can still exercise its supportive functions to face situations that may be perceived as threats (16, 17).

Similarly, it has been evidenced that glucocorticoids and insulin enhance the consumption of fatty foods, which allows us to suppose that the combination of high insulin and cortisol levels can be a powerful inducer of obesity and insulin resistance (18, 19). Currently, it is estimated that there are more than 300 million individuals with obesity, and this figure is expected to increase considerably in the coming years (20). In addition, cardiovascular risk is still one of the leading causes of death in the world.

A meta-analysis encompassing data from 33 studies and over 43,000 participants concluded that elevated stress hormone levels are associated with a higher risk of cardiovascular disease (risk ratio [RR], 1.63; 95% confidence intervals [CIs]: 1.36, 1.97) (21). Discrepancies observed when comparing results from various studies on the relationship between cardiovascular risk and hormones related to anxiety and stress highlight the complexity of this association (22). Part of this complexity lies in the fact that this relationship is influenced by other risk behaviors such as unhealthy diets, physical inactivity, or tobacco use, among others (23). Nevertheless, it is known that hormones associated with states of anxiety, worry, or stress, such as cortisol, can activate pathways leading to increased inflammation and heart rate, as well as platelet alterations, increased sympathetic activity, and reduced parasympathetic activity (24). Therefore, as suggested by Merswolken et al. (25), the evidence linking cortisol level alterations with hypertension, obesity, insulin resistance, hyperlipidemia, and prothrombotic activity suggests that adrenal axis dysfunction may be responsible for the metabolic disturbances that promote cardiovascular disease.

In Colombia, data collected by the Instituto Colombiano de Bienestar Familiar (Colombian Institute of Family Welfare) in the 2015 Colombian Nutrition Survey evidenced that 37.8% of adults between 18 and 64 years of age were overweight and 18.7% were obese (26). Overweight and obesity were more frequent in women than in men (33% vs. 31.1%) (27). There is scientific evidence that describes weight gain, insulin resistance, and hypercortisolism as biochemical factors that could be activated by different life situations, including anxiety and stress (28).

Considering the mental health statistics of the Colombian population and the consistent trend in morbidity and mortality statistics for cardiovascular diseases, the purpose of this study was to evaluate the possible association between trait and state anxiety and altered serum levels of cortisol, lipids, and glycemia, as well as central obesity. These are known factors for cardiovascular risk, which has been scarcely studied in Cartagena and Colombia.

## 2 Methods

### 2.1 Participants

We included 90 participants of both sexes (47 female, 43 male), ranging in age from 25 to 69 years (X̅ = 44.1, SD = 10.9), who met the inclusion criteria. They were selected from a list of 148 faculty members employed full-time. The study sample was drawn from faculty members of a private higher education institution, which comprises five faculties: Education, Social Sciences, Health Sciences, Administrative and Accounting Sciences, Political Sciences, and Engineering, with a student population of approximately 3,300.

As inclusion criteria, we selected faculty members with full-time contracts and more than two years of experience at the university. As exclusion criteria, those receiving medication or dietary regimens that could alter glucose or cortisol levels, diabetes mellitus diagnosis, or adrenal axis dysfunction were not accepted as they could interfere with the results. Each participant provided their written informed consent, and the study was granted ethical approval from the University’s Research Ethics Committee (project code: CIB-CS-2023-01; date of approval: April 18, 2023; Ethics Approval Code Record No. 02/2023). This study followed the guidelines set by the Declaration of Helsinki, and all participants provided written informed consent.

### 2.2 Procedures

#### 2.2.1 Biochemical and anthropometric variables

This quantitative study was conducted with an analytical cross-sectional design, with fasting blood sampling to determine participants’ glycemia and lipid profile levels through spectrophotometric techniques. Participants were instructed to refrain from engaging in physical exercise on the day of sample collection and to abstain from consuming alcoholic beverages for three days prior to venipuncture. Additionally, they were asked to arrive 15 minutes in advance. Dyslipidemia was considered when two or more lipid profile values were altered. Cortisol was measured at 8 AM and 4 PM using a commercial kit based on an electrochemiluminescent immunoassay (Elecsys cortisol II^®^). In all cases, quality controls were used to ensure the validity of the results.

Serum cortisol measurement was preferred in both samples, despite being more invasive than its salivary equivalent, because available evidence has shown that it provides more reliable results and greater discriminatory power (29, 30). Saliva samples, on the other hand, require more stringent collection protocols and exhibit greater variability due to factors such as body weight, caffeine and alcohol intake, antibiotic use, or recent infections (31).

The morning (AM) samples were collected between 8:00 and 9:00 AM, while the evening (PM) samples were taken between 4:00 and 4:30 PM, following the established biosafety protocols for this type of sample. The samples were collected at the clinical laboratory facilities of the university. Serum was immediately separated by centrifugation and stored at 4°C until processing.

Participants’ waist circumference was measured at the midpoint between the last rib and the iliac crest in expiration, using a non-stretch tape measure. To this end, the International Diabetes Federation recommendations for Central and South America were used as cut-off point (>90 cm in men and 80 cm in women) (32).

#### 2.2.2 Psychological variables

To assess trait and state anxiety, we used the Inventory of Trait-State Anxiety (IDARE Spanish version) by Spielberger, C. D., Gorsuch, R. L., Lushene, R., Vagg, P. R., and Jacobs, G. A., which consists of two separate self-assessment scales, each with 20 statements (1983).

This test has several adaptations that ratify its reliability. In the Peruvian adaptation carried out with patients attending weight control, it was found that the Inventory had consistent, objective, and significant psychometric features regarding content, construct, concurrent, and clinical validity. It also has reliability in terms of internal consistency and stability (33). Another study conducted during the COVID-19 pandemic in Peru confirms its psychometric reliability, concluding that it has an adequate factorial structure, measures correctly, and maintains its qualities as an instrument for the evaluation of anxiety (34).

## 3 Data analysis

All data were initially organized using Microsoft Excel and then analyzed using JASP. Descriptive statistics were computed first to summarize the dataset. To examine sex differences, independent samples Student’s t-tests were conducted for morning cortisol (AM), evening cortisol (PM), state anxiety, trait anxiety, and triglycerides. Pearson’s correlation analyses were performed to assess the relationships between morning cortisol, evening cortisol, state anxiety, trait anxiety, and age. Additional Pearson’s correlations were conducted to examine associations between waist circumference and glucose levels.

A Chi-square test was performed to examine the association between abdominal obesity and hyperglycemia, as well as other variables from the lipid profile. Next, a one-way ANOVA was conducted to compare evening cortisol (PM) levels across low, medium, and high state anxiety groups. Finally, a Welch’s t-test was performed to compare triglyceride levels between the presence and absence of state anxiety, as the Brown-Forsythe test indicated a violation of homogeneity of variances.

## 4 Results

The group consisted of 90 participants, with an average age of 44.1 years (SD = 10.9; range = 25–69) and a slight female predominance (47/90; 52.2%).

The internal consistency of the state anxiety items was assessed using McDonald’s Omega (ω) and Cronbach’s Alpha (α). The results indicated high reliability, with ω = 0.94, 95% CI [0.922, 0.958] and α = 0.939, 95% CI [0.918, 0.959].

**Figure 1:**
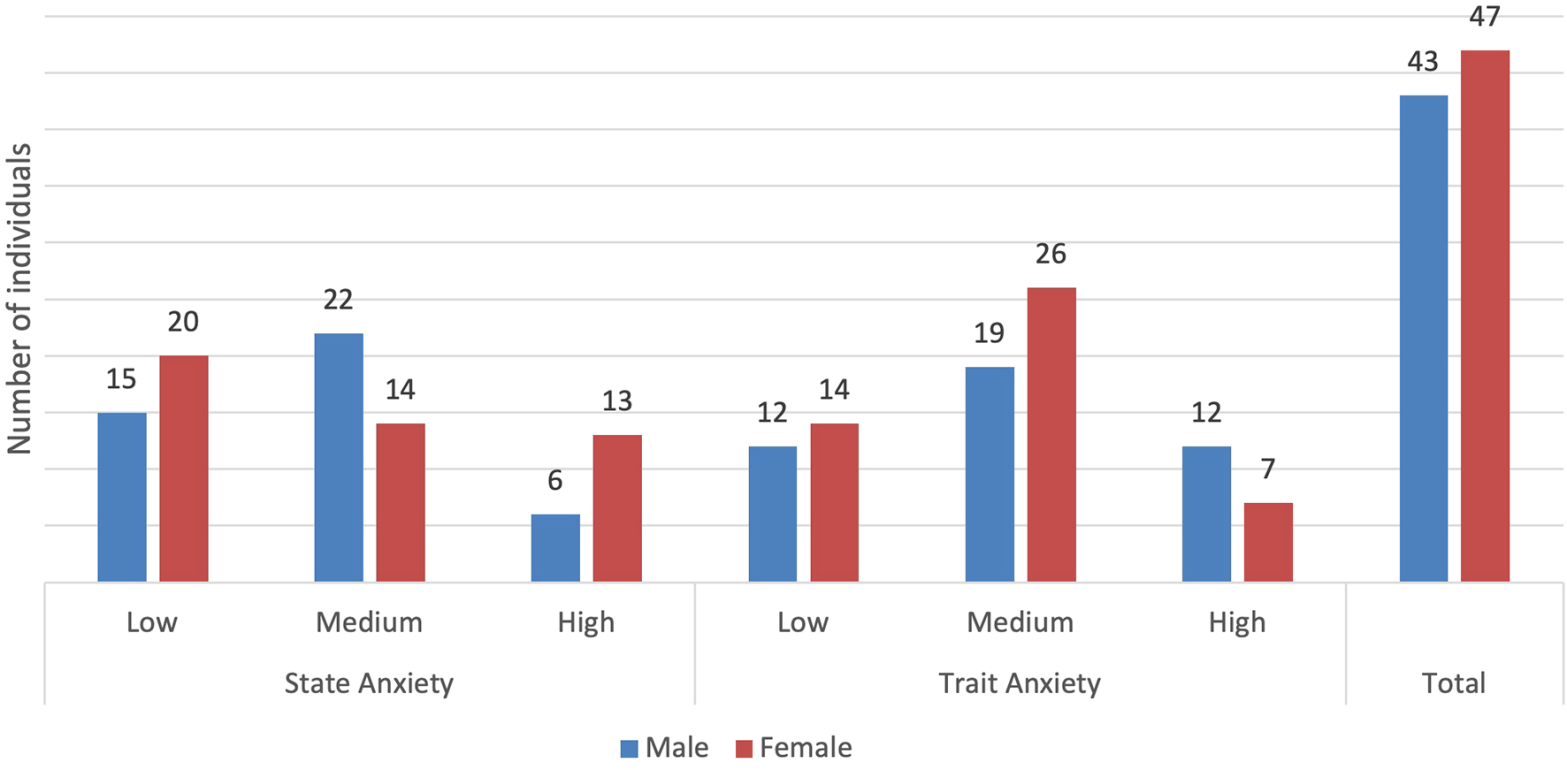
Presence of state and trait anxiety by levels and sex (frequency).

61.1% (55/90) of participants had medium and high levels of state anxiety, and 71.1% (64/90) had medium and high levels of trait anxiety. Women more frequently exhibited high state anxiety and moderate trait anxiety, whereas men more commonly reported moderate trait anxiety (**Figure 1**).

Most participants (94.4%) had AM and PM cortisol levels within normal ranges. The mean AM cortisol was 9.83 µg/dL (SD = 3.63, 95% CI = 9.07–10.59), while the mean PM cortisol was 5.96 µg/dL (SD = 2.74, 95% CI = 5.38–6.53). Cortisol levels were lower in the evening than in the morning for 95.6% of participants (86/90), with AM cortisol ranging from 0.22 to 23.11 µg/dL and PM cortisol from 0.34 to 19.29 µg/dL (**Table 1**). Below-normal levels were observed in 3.3% of participants, and 2.2% had above-normal levels in both measurements. The reference limits were those established by Maji Llinin and Durán Pincay (35).

**Table 1:** AM and PM cortisol levels; total values and values discriminated by state anxiety levels.

	State anxiety level	N	Mean	Typical deviation	Typical error	95% confidence interval for the mean		Minimum	Maximum
						Lower limit	Upper limit		
AM cortisol (µg/dL)	Low	35	9.79	3.47	.59	8.59	10.98	.22	15.83
	Medium	36	9.31	3.01	.52	8.26	10.36	3.12	16.81
	High	19	10.90	4.68	1.07	8.64	13.16	4.83	23.11
	Total	90	9.83	3.63	.38	9.07	10.59	.22	23.11
PM cortisol (µg/dL)	Low	35	5.77	2.33	.39	4.97	6.57	.34	11.69
	Medium	36	**5.13**	1.63	.27	4.58	5.68	1.56	9.74
	High	19	**7.88**	4.05	.93	5.93	9.84	3.61	19.29
	Total	90	5.96	2.74	.29	5.38	6.53	.34	19.29
Cortisol difference (AM−PM) (µg/dL)	Low	35	4.02	2.43	.41	3.18	4.85	-0.12	9.41
	Medium	36	4.18	2.80	.47	3.24	5.13	0.38	11.98
	High	19	3.01	3.93	.90	1.12	4.90	-6.99	10.72
	Total	90	3.87	2.95	.31	3.26	4.49	-6.99	11.98

Values in bold indicate the highest and lowest group means identified in the comparison of evening cortisol levels across state anxiety groups. A one-way ANOVA revealed a statistically significant difference among these means.

**Figure 2:**
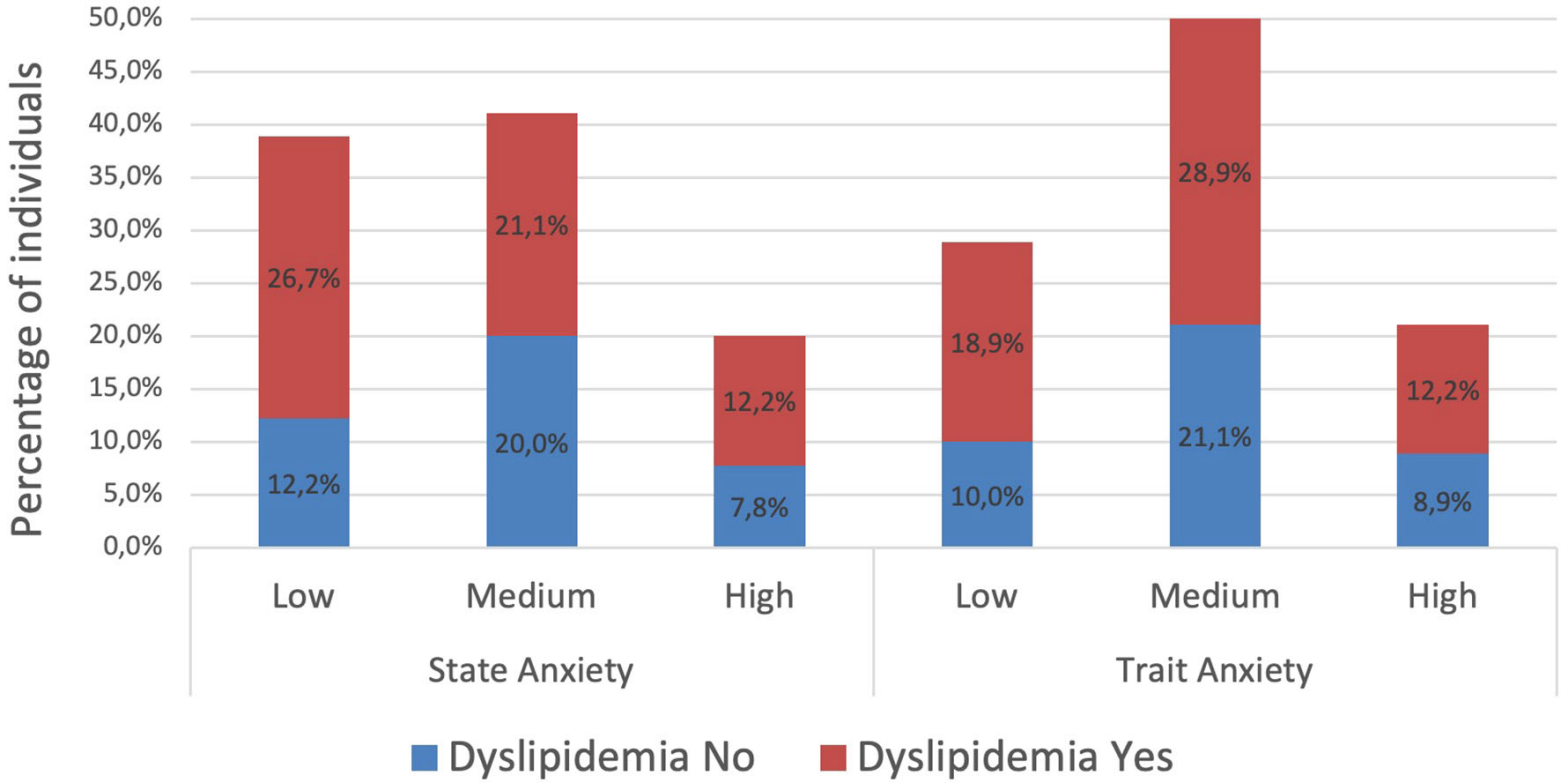
Dyslipidemia (two or more altered parameters) observed in participants divided by level of state or trait anxiety.

The presence of dyslipidemia, defined as the simultaneous alteration of two or more lipid parameters (total cholesterol, triglycerides, and HDL and LDL cholesterol fractions), was observed in 60.4% of the sample (54/90) (**Figure 2**).

Independent t-tests were conducted to compare differences by sex in morning and evening cortisol levels, as well as in state and trait anxiety scores. No significant differences were found between male and female participants in any of these variables, except for triglycerides (t = -2.535, df 89, p= 0.013) (**Table 2**).

**Table 2:** Independent samples t-test.

Variable	t	df	p
AM Cortisol	-0.300	89	0.765
PM Cortisol	-0.432	89	0.667
Trait Anxiety	-1.222	89	0.225
State Anxiety	0.227	89	0.821
Triglycerides	-2.391	89	0.019

Pearson’s correlation analysis showed a significant positive association between state anxiety and PM cortisol levels (r = 0.232, p = 0.028), while no significant relationship was found with AM cortisol concentrations. Additionally, neither cortisol patterns nor anxiety measures (state or trait) were significantly associated with age (**Table 3**).

**Table 3:** Pearson’s correlations between cortisol levels, anxiety scores, and age.

Pearson’s Correlations											
Variable		AM Cortisol		PM Cortisol		State Anxiety		Trait Anxiety		Age	
1. AM Cortisol	Pearson’s r	—									
	p-value	—									
2. PM Cortisol	Pearson’s r	0.603	***	—							
	p-value	<.001		—							
3. State Anxiety	Pearson’s r	0.075		0.232	*	—					
	p-value	0.481		0.028		—					
4. Trait Anxiety	Pearson’s r	-0.033		0.164		0.794	***	—			
	p-value	0.756		0.122		<.001		—			
5. Age	Pearson’s r	-0.005		0.061		0.002		-0.026		—	
	p-value	0.964		0.567		0.982		0.805		—	

*p <.05, ***p <.001.

Of all participants, 68.9% (62/90) had a waist circumference greater than 80 cm in women and 90 cm in men. Pearson’s correlation analysis showed a significant association between waist circumference and fasting glycemia (**Table 4**). Additionally, fasting glycemia was elevated in 11 participants (12.2%).

**Table 4:** Pearson’s correlation between waist circumference and glycemia.

Pearson’s Correlations					
Variable		Waist Circumference		Glycemia	
1. Waist Circumference	Pearson’s r	—			
	p-value	—			
2. Glycemia	Pearson’s r	0.319	***	—	
	p-value	<.002		—	

***p <.001.

Using a Chi-square test, the presence of abdominal obesity showed a significant association with hyperglycemia (p = 0.010, df = 2), but not with the other variables from the lipid profile.

A one-way ANOVA was conducted to examine differences in evening cortisol levels across state anxiety levels (low, medium, and high). The results indicated a significant effect of state anxiety on evening cortisol levels, F(2, 87) = 7.336, p = 0.001. Mean comparisons showed that individuals in the high state anxiety group had the highest evening cortisol levels (X̅ = 7.88, SD = 4.05), followed by those in the low state anxiety group (X̅ = 5.77, SD = 2.37), while the moderate state anxiety group had the lowest levels (X̅ = 5.13, SD = 1.63).

Similarly, the one-way ANOVA revealed a statistically significant difference in mean PM cortisol levels among state anxiety groups (F(2, 87) = 7.336, p = 0.001). The high state anxiety group had the highest mean cortisol levels (X̅ = 7.88 µg/dL), followed by the low state anxiety group (X̅ = 5.77 µg/dL) and the moderate state anxiety group, which had the lowest mean levels (X̅ = 5.13 µg/dL).

An independent samples Welch’s t-test was conducted to compare the concentrations of each evaluated lipid fraction based on the absence (low levels) and presence (medium and high levels) of state anxiety. Welch’s t-test was preferred since the Brown-Forsythe test indicated a violation of homogeneity of variances when using Student’s t-test in multiple comparisons. A significant difference was found only for triglyceride levels between the absence and presence groups. The Welch’s t-test indicated a statistically significant difference, t(87.999) = -2.244, p = 0.027. The presence group had a higher mean triglyceride level (X̅ = 129.58, SD = 62.32) compared to the absence group (X̅ = 105.53, SD = 39.32).

## 5 Discussion

No significant differences were observed between sexes for any type of anxiety, although higher levels may be expected in women, as it has been evidenced in several epidemiological studies in which anxiety has a marked influence on females (8). This effect, however, could be more pronounced with larger samples.

It was found that substantial cortisol alterations in the afternoon were associated with state anxiety, which highlights the importance of studying the presence of other medical and mental health conditions due to the high correlation among anxiety, other mental health conditions, and physical diseases (3). Our findings also support the role that mental diseases, and particularly anxiety, may play in the onset or prevalence of medical conditions via cortisol alterations (36). In the following paragraphs, our study results are contrasted with those of previous studies that support the conclusions mentioned above.

Anxiety as an assessment measure for psychological dysfunction is particularly relevant, not only because it is the most prevalent mental disorder, but also because it has regularly been found as a comorbidity of other mental health conditions. For example, Mielimąka et al. (37) found that 69% of patients with personality disorders had state anxiety and 64.7% had trait anxiety. For this reason, personality disorders are a primary condition to take into account in the mental health assessment and intervention of anxious patients.

The results of studies with different designs focused on social anxiety are consistent with our findings and conclusions about the relevance of anxiety as a risk factor, especially in stressful situations. Mirete et al. (38) studied the increase in salivary cortisol levels in individuals with high and low levels of social anxiety in the face of stressful situations and found that in both groups, cortisol levels varied under stress conditions, although the response was significantly higher in individuals with high anxiety levels.

Our findings showed a significant relationship between the presence of state anxiety and the increase in evening cortisol concentrations. In addition, there is a tendency toward an inverse relationship with a reduced difference between AM and PM cortisol levels in the groups with higher levels of trait anxiety. This is consistent with the evidence showing a relationship between circadian rhythm maintenance and health and points to the importance of assessing cortisol levels at these two times to conduct a comprehensive monitoring of the psychological and biological state of individuals (39).

The relationship between PM cortisol levels and state anxiety has also important implications for the development of anxiety management strategies. In particular, the possible dysregulation of the HPA axis in response to stress, along with the association between elevated PM cortisol levels, greater emotional reactivity, and a lower capacity for stress recovery (40, 41), suggests the need to explore interventions focused on stress regulation, lifestyle, and sleep. These strategies may contribute to better physiological and emotional adaptation to stress, as well as a reduction in state anxiety.

In this regard, Cognitive Behavioral Therapy (CBT) has shown promising effects in regulating cortisol levels and sleep patterns across various populations. In older adults with generalized anxiety disorder, combining CBT with SSRI treatment significantly reduced peak cortisol levels compared to SSRI use alone (42). Similarly, pregnant women receiving CBT for stress management experienced reductions in both hair cortisol levels and psychological stress (43). In heart failure patients, although CBT for insomnia did not directly affect cortisol levels, improvements in sleep-related symptoms were associated with an increased day-to-night cortisol ratio (44).

Mindfulness-based interventions have also demonstrated positive effects on stress regulation and cortisol levels. Long-term meditators exhibit reduced morning cortisol levels, while novices who completed an eight-week Mindfulness-Based Stress Reduction (MBSR) program showed lower cortisol levels and improved sleep quality (45). In cancer patients, MBSR helped normalize cortisol levels by increasing low baseline levels and reducing elevated ones (46). Even short programs, such as a four-day mindfulness meditation training for medical students, have been shown to significantly reduce serum cortisol levels (47).

Additionally, physical activity plays a key role in stress regulation and cortisol levels. Regular aerobic exercise has been associated with more adaptive cortisol secretion patterns and lower stress reactivity (48). Moreover, sleep hygiene interventions, such as Cognitive Behavioral Therapy for Insomnia (CBT-I), have shown positive effects in normalizing circadian cortisol rhythms and reducing state anxiety (49). Since both sleep and physical activity directly influence HPA axis function, integrating these strategies into stress management programs could enhance the effects of other psychological interventions and improve long-term emotional regulation.

Given the significant association between cortisol and anxiety in educators, implementing evidence-based stress reduction programs specifically tailored to this population could be instrumental in promoting faculty well-being and preventing burnout. Wagner and Pearcey (50) found that different stress reduction activities had varying effects on salivary cortisol levels in educators. Specifically, meditation and yoga significantly reduced cortisol levels 30 minutes post-activity, while aerobic exercise paradoxically increased cortisol levels, suggesting that certain interventions may be more effective in mitigating physiological stress responses. Similarly, von der Embse et al. (51) conducted a systematic review of teacher stress interventions, concluding that mindfulness-based, behavioral, and cognitive-behavioral approaches were the most effective at reducing educator stress and improving occupational outcomes. These findings align with our results, emphasizing the need for targeted interventions that address both the psychological and physiological aspects of anxiety among faculty members.

Although the cross-sectional design of this study does not allow for the establishment of cause-and-effect relationships, it is important to consider that the relationship between cortisol levels and mental states could be bidirectional, as stated by Qin et al. (52). This situation once again highlights the complexity that must be acknowledged when studying human beings, given that the presence of anxiety triggers cortisol release and alters cardiovascular health, including body weight and fat distribution, which in turn also influences anxiety levels. Longitudinal studies under more controlled conditions could be useful in clarifying the predominant directionality.

The evidence obtained in a group of patients with depression showed that alterations in the circadian rhythm of cortisol were more frequent in the group that also had anxiety, which suggests that these values should be used as one of the markers of depression and anxiety (53) and should be taken into account when assessing cardiovascular risk related to excess cortisol and rhythm alterations (54).

Other studies have shown that cortisol levels are positively associated with negative thoughts, and the intensity of the latter affects the onset of anxiety and depression. This justifies the need to include these values in screening this type of disorder (55).

An experimental study with young individuals with high and low social anxiety evidenced a positive correlation for cortisol increase as a consequence of a greater stress factor in the group with high social anxiety, which implies a greater reactivity of the adrenocortical axis under these conditions (38). In fact, these alterations could be associated with chronic diseases, such as cancer, and it has been suggested that glucocorticoid levels may favor tumor growth and be related to psychological states (56).

In 2009, Veen et al. published the results of a study of 72 adults with depression or anxiety, in which they concluded that high baseline cortisol concentrations and decreased circadian variability of cortisol were associated with less favorable lipid profiles, in particular due to decreased high-density lipoprotein–cholesterol. This supported the hypothesis of a verifiable relationship between affective disorders and cardiovascular disease mediated by the adrenal axis (57).

More recently, Anni et al. (58) found that stressful life events were significantly associated with altered lipid patterns and elevated serum triglyceride levels in middle-aged Korean men, highlighting the potential metabolic impact of psychological stress. Other authors, on the contrary, have reported an increase in cholesterol and no association with triglycerides (59). These discrepancies may be attributed to the approach used to define the presence of anxiety, an aspect that should be standardized and validated through its application in population studies, given that different stressors could trigger various forms of adrenal axis activation and, consequently, different impacts on serum biochemical levels.

Studies exploring suicidal ideation, acute coronary syndrome, and cortisol levels have shown that the prediction of suicide could be improved by assessing serum cortisol in its acute phase, which could also impact morbidity and mortality in patients with heart conditions, which is especially relevant in cardiovascular clinics (60). Among young people, depression was associated with increased cortisol in saliva and serum triglycerides, which may also increase their cardiovascular risk (61).

Regarding the presence of state and trait anxiety in our study group, we found medium and high levels of 61.5% and 71.4% for state and trait anxiety, respectively, with no difference between the sexes. Previous studies in healthcare workers had shown moderate or severe anxiety levels in 26.1% of participants; these values, which are very low when compared with our findings, reported an association with dyslipidemia (PR = 2.07; 95% CI = 1.74–2.45). This differs from our results, although there is a similarity regarding the high frequency of dyslipidemia (54.8%) (62).

In addition, it is known that anxiety-related disorders have been linked to alterations in lipid metabolism due to chronic deregulation of the adrenal axis and the action of the hormones related to this state, such as catecholamines and gamma-aminobutyric acid. Cholesterol, the main source of steroid hormones, plays a very important role in this axis, including circadian rhythm, stress, and neuropsychiatric disorders (63).

Based on the results obtained, it is possible to suggest that state anxiety may be associated with the sustained elevation of cortisol levels. No studies were found that specifically examined the impact of each type of anxiety on the expected decline in serum cortisol concentrations in the afternoon. However, our findings align with the evidence reported by Mohd Azmi et al. (54), which indicates that cortisol regulates the circadian rhythm and is linked to cardiovascular disease.

In cases of sleep disorders, jet lag, or mental conditions such as stress or anxiety, circadian dysfunction can be explained by the loss of negative feedback on the adrenal axis, which could account for the sustained increase in cortisol levels in the afternoon among individuals with higher anxiety levels. Additionally, the greater incidence of circadian cortisol alterations observed in patients with depression accompanied by anxiety (53) further supports this association.

In contrast to our findings, a study conducted in 2013 with a sample of 142 individuals showed that total cholesterol and high-density lipoprotein levels were higher in the group with higher levels of anxiety, but no differences were found for triglyceride, high-density lipoprotein, and platelets (59).

In addition to the above, a study conducted in patients with personality disorders also showed a positive relationship between cortisol and testosterone levels, which seems to suggest that sex could be related to alterations in the adrenal axis (64). Previous studies have shown that gender perception was related to different biological responses to an intervention aimed at improving regulation, suggesting that gender roles may influence the hypothalamic–pituitary–adrenal axis function during episodes of acute stress (65).

Multiple studies agree on the effect of negative experiences in childhood on alterations of the adrenal axis (66, 67), and show varying degrees of association with the onset of depression and anxiety, establishing a link between these experiences and adult mental health (68). In contrast to these findings, our results showed no differences between sexes, suggesting that it may be necessary to study this aspect in depth and include other variables, improving the approach to gender perceptions and childhood experiences that could affect adulthood.

Currently, hybrid models have been proposed to promptly identify heart diseases (69), accurately and reliably classify lung cancer types (70), and, by leveraging the strengths of combined models, integrate complex aspects such as space-time relationships, as proposed by Ali et al. (71). These models would be particularly useful for establishing more precise relationships between variables that follow a circadian rhythm, such as cortisol.

One limitation of our study is that it did not address aspects related to participants’ previous experiences, including their childhood and adolescence, which could have provided robust follow-up of these disorders. In addition, lifestyle assessments that could positively or negatively affect the associations observed were not included. However, it is worth mentioning that participants who followed special diets or used medications that could affect the results were excluded.

## 6 Conclusion

The presence of abdominal obesity and lipid profile alterations were frequent, regardless of age or sex, as well as medium and high levels of trait and state anxiety.

Our study results showed cortisol levels within the reference values in most of the participants, although alterations in the afternoon were also observed. These were associated with state anxiety, which highlights the importance of studying the presence of other medical and mental conditions due to the high correlation between anxiety, other mental conditions, and physical diseases. Our findings also support the role that mental diseases, particularly anxiety, may play in the onset of medical conditions via cortisol alterations.

Furthermore, this study confirms the association between lipids, cortisol and anxiety, which has been reported by a growing number of studies, which highlights the potential value of measuring this hormone during medical examinations, preventive medicine, and mental health interventions, either through psychopharmacological and/or psychotherapeutic treatments.

Our findings, together with those of similar studies following the same lines, support the statement that mental health interventions, such as psychoeducation, training in stress management, problem-solving skills, social skills, and emotional regulation, among others, are important for preventing diseases. In the case of anxiety, interventions aimed at its mitigation or prevention may also help prevent cardiovascular diseases (and others) resulting from the sum of lipid and glycemia alterations and consistently high cortisol levels. The presence of state anxiety was found to be the type of anxiety most associated with increased evening cortisol levels and triglyceride levels.

It is relevant to continue exploring the relationships between psychological and biochemical variables, as they could serve as important inputs for developing AI-supported programs that enhance the diagnosis, monitoring, and outcomes of physical and mental health treatments.

The differences observed in other studies regarding the association found between the levels of anxiety, cortisol, and lipids could be due, in part, to the high frequency of alterations in the biochemical variables in most of the study population. This reflects the importance of approaching health from a global perspective that integrates biological and psychological factors to contribute to the present and future well-being of the population.

## Data Availability

The raw data supporting the conclusions of this article will be made available by the authors, without undue reservation.
